# Participatory adaptation of mental health screening tools for school-aged children: a qualitative study in Slovakia

**DOI:** 10.3389/fpsyg.2026.1649469

**Published:** 2026-03-25

**Authors:** Lenka Janik Blaskova, Kornelia Durikova

**Affiliations:** 1 Institute of Applied Psychology, Faculty of Social and Economic Sciences, Comenius University in Bratislava, Bratislava, Slovakia; 2 Koalicia skol za dusevne zdravie, Liga za dusevne zdravie, Bratislava, Slovakia

**Keywords:** children and young people, cultural validation, mental health screening, participatory research, research-practice partnership, schools, Slovakia, focus groups

## Abstract

**Introduction:**

Mental health difficulties affect many school-aged children worldwide, yet culturally validated tools for early screening remain limited. This study reports the initial phase of adapting mental health screening tools for Slovak schools, focusing on translation, cultural adaptation, and children’s perspectives to enhance relevance and comprehension.

**Methods:**

Using a research-practice partnership model, we conducted participatory focus group discussions to explore children’s understanding of the *Me and My Feelings*, *Student Resilience Survey*, and *Short Warwick-Edinburgh Mental Wellbeing Scale*. Participants (*N* = 108) from three school levels provided feedback on wording, examples, and formatting, which informed iterative revisions and assessed the acceptability and feasibility of the Slovak versions.

**Results:**

Children struggled with abstract concepts and culturally specific items. Revisions improved linguistic, semantic, and operational equivalence, clarified response formats, and incorporated more relatable examples. Variations in prior experiences with self-report questionnaires, as well as observed challenges in completing items, highlighted the accessibility and feasibility considerations for younger pupils, non-native Slovak speakers, and children with special educational needs.

**Conclusion:**

Participatory methods and research-practice partnerships enhance clarity, cultural relevance, and practical usability of screening tools. These adaptations provide a foundation for subsequent psychometric validation and context-sensitive implementation in Slovakia and similar educational contexts.

## 1 Introduction

School-aged children face considerable risks of mental health difficulties worldwide. Recent global estimates suggest that approximately one in seven 5–19 year olds, around 14%, experience mental health challenges (Institute for Health Metrics and Evaluation, 2025). These difficulties are associated with poorer long-term developmental outcomes, including adverse mental and physical health (Otto et al., 2021), and can negatively affect academic performance and social functioning (Smith et al., 2021). Conversely, positive mental wellbeing supports prosocial behaviours, resilience, and a healthy self-concept (Dempsey et al., 2024).

Early identification of mental health concerns is crucial, and screening tools play an important role in detecting potential risks among children and young people. Some tools assess general emotional and behavioural difficulties (e.g., the Strengths and Difficulties Questionnaire; Goodman, 1997), while others target specific domains such as resilience or wellbeing (e.g., Lereya et al., 2016). Schools, due to their regular and universal contact with children, represent a strategic setting for the early identification and monitoring of mental health difficulties and for supporting timely intervention (Deighton et al., 2013; Lereya et al., 2016).

Most mental health screening tools and programmes originate in English-speaking countries, where awareness and resources are often greater than in other regions. The seminal guidelines of Guillemin et al. (1993) continue to underpin cross-cultural adaptation practices and are widely cited in contemporary studies (Cruchinho et al., 2024; Fekar Gharamaleki and Fathipour-Azar, 2025). Many countries adopt English-based tools due to limited local expertise or resources to develop their own measures (Rahman et al., 2003). The use of standardised instruments across countries facilitates multinational comparisons and monitoring of global mental health trends, while also supporting context-specific adaptations. Examples include the World Health Organisation’s (WHO) mental health measures (e.g., WHO-Five Well-Being Index, 2004) and the Health Behaviour in School-aged Children (HBSC) study [World Health Organisation (WHO), 2025].

This study exemplifies such international collaboration. The Slovak charity, Liga za dusevne zdravie [the League for Mental Health], works with the Anna Freud in the United Kingdom, which provides consultancy and resource sharing to enhance mental health initiatives in Slovak schools. The collaboration includes adaptation of the Mentally Healthy Schools Programme, which involves regular screening of children’s mental health. The Slovak adaptation is called Koalicia skol za dusevne zdravie [the Coalition of Schools for Mental Health], and its associated screening tool is known as Pohodomer. As part of the adaptation process, we consulted the original tool authors and the Anna Freud Centre to ensure conceptual integrity. This study focuses on the translation and cultural adaptation of selected Pohodomer instruments, specifically the Me and My Feelings (Deighton et al., 2013), Student Resilience Survey (Lereya et al., 2016), and the Short Warwick-Edinburgh Mental Wellbeing Scale (SWEMWBS; Stewart-Brown et al., 2009).

### 1.1 Translation and cultural adaptation of mental health screening tools

Translation and cross-cultural adaptation of English-based tools are essential to ensure validity, cultural relevance, and appropriateness for the target population. Peña (2007) identifies key forms of equivalence to consider: linguistic, functional, cultural, and metric. Herdman et al. (1998) propose a complementary framework encompassing conceptual, item, semantic, operational, and measurement equivalences. Recent studies applying these frameworks emphasise participatory approaches and pretesting with children and young people to enhance cultural relevance and comprehension (Cruchinho et al., 2024; Fekar Gharamaleki and Fathipour-Azar, 2025; Verde-Avalos et al., 2025). These models guide the adaptation processes in child development (Peña, 2007) and quality-of-life research (Herdman et al., 1998), making them relevant to this study.

Conceptual equivalence refers to the extent to which the constructs have the same meaning and relevance across cultures and should be examined prior to translation, as cultural differences may influence how constructs are understood (Herdman et al., 1998). Linguistic or semantic equivalence involves accurate translation, typically through forward and back translation and expert consultation to resolve discrepancies (Cruchinho et al., 2024; Herdman et al., 1998; Peña, 2007).

Functional equivalence ensures that the tool measures the same construct in the new culture, while cultural equivalence concerns how items and responses are interpreted (Herdman et al., 1998; Peña, 2007). Operational equivalence addresses administration procedures, instructions, and formats (Herdman et al., 1998). Measurement equivalence refers to psychometric properties such as reliability and validity.

Metric equivalence (Peña, 2007), referring to similarity in item difficulty across cultures, is less relevant here because the selected tools assess experiences rather than performance (Herdman et al., 1998). Similarly, establishing full psychometric equivalence is most critical when cross-cultural comparisons are intended (Peña, 2007). In the present study, the tools are used to inform individual schools about risk and protective factors affecting the psychosocial experiences of children and young people, rather than cross-national comparisons. Nonetheless, formal psychometric evaluation of the Slovak versions may be considered in future research.

Guidelines for translation and cultural adaptation vary. The World Health Organisation (WHO) recommends forward translation, expert review, back-translation, and pretesting with the target population (Snider and Schafer, 2018). Cruchinho et al. (2024) propose an eight-step process including translation, harmonisation, and field testing. Sousa and Rojjanasrirat (2011) outline a seven-step process, the first five steps focusing on wording and cultural adaptation. In a systematic review of adaptations of the Cognitive Emotion Regulation Questionnaire, Fekar Gharamaleki and Fathipour-Azar (2025) demonstrated the importance of transparent translation and cultural adaptation procedures; however, three of the 13 destination countries reviewed did not report sufficient methodological details.

Translation, cultural adaptation, and validation are time- and resource-intensive processes requiring rigorous methodology and involvement of experts and the target population (e.g., Cruchinho et al., 2024; Snider and Schafer, 2018; Sousa and Rojjanasrirat; 2011). As a result, researchers often build on existing translations and focus primarily on psychometric validation (e.g., Ronzón-Tirado et al., 2019; Verde-Avalos et al., 2025). The present study concentrates on the earlier stages of translation and cultural adaption, given resource constraints and the primary goal of informing individual schools.

Levels of cross-cultural validation depend on the relationship between the original and target populations, ranging from “no change in culture” and “immigrants in the original country” to “different country but same language” to “different country and language” (Cruchinho et al., 2024; Guillemin et al., 1993). Potential sources of bias include conceptual differences, item relevance, and administration methods (Cruchinho et al., 2024).

Contemporary studies continue to highlight context-sensitive adaptations of English-based instruments. Granat et al. (2022) examined item clarity and comprehensibility in a Swedish healthcare adaptation of a self-efficacy in palliative care scale. Mao et al. (2021, p. 99) identified conceptual differences between Chinese and Western quality-of-life measures, noting the inclusion of traditional Chinese medicine concepts such as “weather adaption” and “spirit,” which are absent in Western instruments. These examples illustrate that although, foundational guidelines such as Guillemin et al. (1993) remain highly relevant, participatory and context-sensitive approaches are increasingly emphasised. Similarly considerations apply in Slovakia, where school-based mental health initiatives remain relatively recent and inconsistently implemented.

### 1.2 Research-practice partnership

Drawing on the shared goal of improving education and mental health in Slovak schools, this study emerged from a research-practice partnership between university researchers and the Slovak national mental health charity, Liga za dusevne zdravie. Such partnerships aim to bridge the gap between research and practice and are recognised for their potential to foster both target interventions and systemic change (McGeown and Sjölund, 2025). Our work adds to examples from European countries including Sweden, Norway, Germany, England, and Scotland, highlighted by McGeown and Sjölund (2025).

Building on previous collaborations (e. g., Janik Blaskova and Winter, 2025, this partnership was a natural progression. Liga za dusevne zdravie [the League for Mental Health] launched Koalicia skol za dusevne zdravie [the Coalition of Schools for Mental Health] to work directly with schools to promote wellbeing and prevent mental health difficulties among pupils, students, and staff. For a small annual fee, member schools gain access to mental health activities, a regional coordinator, and detailed consultation on their Pohodomer results. To date, the League has gathered data from approximately 15,000 pupils and students and 1,500 teaching staff. In some schools, longitudinal data have been collected over multiple years, enabling monitoring of longer-term trends.

Prior informal feedback and observations from practice have indicated strong face validity of the Pohodomer. Schools report that it captures relevant issues and clarifies areas of concern. The data help schools identify mental health challenges and guide the development of psychologically safe environments.

Despite its widespread use, Pohodomer has not yet undergone formal cultural validation. Given increasing national interest in school-based mental health screening, the Ministry of Education, Research, Development and Youth and affiliated organisations such as the Research Institute of Child Psychology and Pathopsychology (VUDPaP), the National Institute for Education and Youth (NIVAM), and the State School Inspection have expressed interest in identifying or developing similar tools. This has further motivated Liga za dusevne zdravie to strengthen the methodological rigour and psychometric quality of the instrument. Table 1 summarises selected tools and their current use within Pohodomer.

**Table 1:** Overview of tools used in Pohodomer by educational stage, age group, and source.

Tool	Authors	Number of items	School level (age range in years)		
			Primary school (8–11 y/o)	Lower secondary (11–15 y/o)	Upper secondary (15–19 y/o)
Me and My Feelings	Deighton et al. (2013)	16	✓		
Student Resilience Survey	Lereya et al. (2016)	38	✓	✓	✓
Short Warwick–Edinburgh Mental Wellbeing Scale	Stewart-Brown et al. (2009), Warwick-Edinburgh Mental Well-Being Scale (2008)	7		✓	✓

Primary school, Years 3–4, Age 8–11 years; Lower secondary school, Years 5–9, Age 11–15 years; Upper secondary school, Years 10–14, Age 15–19 years.

The research-practice partnership team shared a commitment to including children and young people in the cultural adaptation process. Accordingly, we extended the participatory approach of research-practice model to the stakeholder groups targeted by Pohodomer. Participatory approaches have gained prominence over the past four decades (Duea et al., 2022), and consultation with target populations is recognised as a core principle [International Collaboration for Participatory Health Research (ICPHR), 2013].

### 1.3 Study aims and approach

This study examines existing Slovak translations of selected tools used to screen mental health experiences of children and young people in schools. Feedback from administrators highlighted limitations in item wording, prompting a systematic review of the translations. The aim was to revise the wording and enhance linguistic and cultural relevance in order to improve data quality. Following adaptation, the tools may also be relevant to organisations such as the Slovak Ministry of Education, VUDPaP, NIVAM, and the State School Inspection.

The study adopts a qualitative approach, actively involving children and young people from schools outside the Koalicia skol za dusevne zdravie to explore how they interpret and relate to the questionnaire items. This approach is consistent with qualitative methods recommended in instrument adaptation research (Guillemin et al., 1993; Peña, 2007; Sousa and Rojjanasrirat, 2011). Focus group discussions were conducted to identify potentially problematic items, instructions, and response options, and to generate alternative wording that is clearer and more meaningful.

The present study has three key objectives:

1. To evaluate the acceptability and feasibility of selected mental health questionnaires when administered to children and young people.
2. To examine how children and young people interpret and understand questionnaire items and response options.
3. To refine the linguistic and cultural adaptation of the selected tools based on empirical findings from the adaptation process.

By achieving these objectives, we aim to better align the tools with Slovakia’s linguistic and cultural context and support school-based mental health initiatives through improved data quality.

## 2 Materials and methods

### 2.1 Methodological approaches

This study employed a qualitative approach to achieve its objectives. It followed established guidelines from the WHO and other researchers (e.g., Cruchinho et al., 2024; Zhao et al., 2024), while adjusting the sequence of steps as the tools had already been translated into Slovak and were in use by Koalicia skol za dusevne zdravie in Slovakia.

Building on the revised Brislin's (1970) translation model, we engaged in group discussions with bilingual experts in the field of psychology and education (Jones et al., 2001). We adopted a symmetrical translation approach, which prioritises conveying culturally relevant meaning rather than literal equivalence (Cruchinho et al., 2024; Sousa and Rojjanasrirat, 2011). This approach was applied when the original meaning of items did not fully align with their literal translations or when direct translation was not feasible due to cultural differences. The symmetrical approach was consistent with the study’s participatory methodology, as children and young people were consulted to develop a version of the instrument that was meaningful for them. Their suggestions were incorporated into the revised Slovak versions prior to back-translation.

### 2.2 Research population

In line with the participatory approach, this study sought representatives from the target groups of the selected tools. A convenience sampling strategy was used to recruit participating schools, beginning with schools where the research team had established contacts with teachers acting as gatekeepers. Schools were selected based on prior collaboration and willingness to participate. Participants were recruited specifically for this qualitative study; the questionnaires were not administered at the whole school level.

Efforts were made to include a range of age groups across school levels and to capture variation at the upper secondary level. Participants were recruited from a gymnasium, which provides a broad academic education aimed at university entry, and a secondary industrial school of engineering, which focuses on technical and vocational training. A key inclusion criterion was that participants had no prior experience with the screening tools.

Teachers informed children and young people about the study, emphasising voluntary participation,. and distributed information sheets to carers. Informed consent was obtained from carers and from young people over 16 years of age, while informed assent was obtained from younger participants.

Participants were recruited from two locations in Slovakia. Site 1 is a small urban city in the south of the country, while Site 2 is a mid-sized urban city in the northwest. Locations outside the capital, Bratislava, were intentionally selected, as mental health and wellbeing activities, including screening and research activities, are more established there than in other regions. This approach aimed to include participants who may better reflect the broader Slovak population. The names of the participating cities have been anonymised to protect confidentiality.

Table 2 presents the sociodemographic characteristics of the sample, including location, school level, and the screening tools completed and discussed.

**Table 2:** Sociodemographic characteristics of participants by location, school level, and tools review.

School level and years	Typical age (Years)	Location	Total (N)	Gender		Selected tools		
				Girls	Boys	M&MF	SRS	SWEMWBS
Primary								
Year 3	8–9	S1-S	23	13	10	✓	✓	
Year 4	9–10	S2-NW	17	11	6	✓	✓	
Lower secondary								
Year 6	11–12	S1-S	17	7	10		✓	✓
Year 8	13–14	S2-NW	16	8	8		✓	✓
Upper secondary								
Year 10 (Gymnasium)	16–17	S1-S	21	15	6		✓	✓
Year 10 (secondary industrial school of engineering)	16–17	S2-NW	14	2	12		✓	✓
		Total (N)	108	56	52	40	108	68

M&MF, Me and My Feelings; SRS, Student Resilience Survey; SWEMWBS, Short Warwick–Edinburgh Mental Wellbeing Scale. S1-S, Site 1, Southern Slovakia; S2-NW, Site 2, Northwestern Slovakia.

### 2.3 Research tools

This study drew on a multiple qualitative data sources across three phases to inform the translation and cultural adaptation of existing Slovak versions of selected mental health questionnaires. Table 3 provides an overview of each phase, including input data, parties involved, and key outputs.

**Table 3:** Overview of the three-phase process of tool review, validation, and refinement.

Phase number and title	Input	Parties involved	Output
Phase 1: Review and revision of existing versions	1.1 Original English versions 1.2 Existing Slovak translations of the selected questionnaires in Pohodomer 1.0 1.3 Researcher notes from comparing original tools and Pohodomer 1.0 1.4 Notes from research-practice partnership meetings	Researchers Koalicia skol za dusevne zdravie [Coalition of Schools for Mental Health] Pohodomer 1.0 administrators	List of items to review with children and young people Script for meetings and discussions with children and young people
Phase 2: Participant feedback and group discussion	2.1 Existing Slovak translations of the selected questionnaires in Pohodomer 1.0 2.2 Questions from children and young people during filling in the questionnaires 2.3 Researchers notes when administering the selected tools 2.4 Focus group discussions (verbatim transcripts of audio-recordings)	Children and young people Researchers Koalicia skol za dusevne zdravie	Participants’ experiences in filling in the selected tools Suggestions for adaptation of some items from the existing list Confirmations of some of the items New items to discuss
Phase 3: Tool refinement	3.1 Revised items in Slovak 3.2 Back-translation of the revised Pohodomer 1.1 3.3 Researcher notes from discussing the back translation with the translator 3.4 Consultation within the Research-practice partnership team 3.5 Changes outlined for consideration by the original authors of the tools and the Anna Freud Centre	Translator Researchers Koalicia skol za dusevne zdravie Original authors Anna Freud Centre	Back-translated Pohodomer 1.1 Document detailing the proposed changes after back-translation and analysis of focus groups Acknowledgment by the original authors and the Anna Freud Centre Pohodomer 2.0 New notes for Pohodomer 2.0. administrator manual

#### 2.3.1 Phase 1: review and revision of existing versions

Key data sources in this phase included the original English versions of the questionnaires, existing Slovak translations, and researcher notes from collaborative sessions with the research-practice partnership team. Feedback was also gathered from representatives of Koalicia skol za dusevne zdravie, based on their experience administering the tools. This phase resulted in a list of items identified for further review and adaptation.

#### 2.3.2 Phase 2: participant feedback and focus group discussions

In this phase, qualitative data were collected. Children and young people completed the selected Slovak questionnaires individually and anonymously within their respective year groups. Participants were invited to annotate or raise questions about any unclear items. These annotations and questions, together with researcher observations and notes recorded during and after questionnaire administration, informed the identification of potential revisions.

Immediately following questionnaire completion, semi-structured focus groups were conducted (Guillemin et al., 1993; Peña, 2007; Schoua-Glusberg and Villar, 2014; Snider and Schafer, 2018; Sousa and Rojjanasrirat, 2011). These discussions explored participants’ interpretations of unclear items, perceived cultural relevance, and emotional resonance. An interview guide was developed to ensure consistency across groups and included pre-identified items while allowing flexibility to explore additional issues raised by participants.

Researchers moderated the discussions to ensure a safe and open environment and took detailed notes to capture contextual factors and non-verbal cues. In Years 1–4 of primary school, class teachers were present, as pupils are typically taught all subjects by the teacher and have established relationships with them. At higher grade levels, researchers were alone with students during questionnaire completion and discussions to support confidentiality and psychological safety. All sessions were audio-recorded using an offline voice recorder and transcribed verbatim for analysis.

#### 2.3.3 Phase 3: tool refinement

In the final phase, revised Slovak questionnaire items and their back-translations into English were reviewed by the first author and an independent translator. Discrepancies between the original and back-translated versions were discussed and resolved. A summary of proposed changes was prepared, and consulted with the research-practice partnership team. Selected revisions were also flagged for consultation with the original tool authors and the Anna Freud Centre to ensure conceptual integrity and transparency. This phase resulted in the final version of the questionnaire, Pohodomer 2.0.

### 2.4 Research analysis

Qualitative data, including the original and translated versions of questionnaires, researcher notes, focus group transcripts, were analysed using complementary methods to ensure a comprehensive understanding of the translation and cultural adaptation process.

First, an item-by-item comparative review of the original and translated questionnaires was conducted in collaboration with the translator and the research-practice partnership team. This process assessed linguistic accuracy, conceptual consistency, clarity, and cultural relevance.

Second, focus group transcripts were analysed using a descriptive qualitative approach to capture participants’ interpretations, understanding, and emotional responses to specific questionnaire items (Peña, 2007; Schoua-Glusberg and Villar, 2014; Snider and Schafer, 2018). Transcripts were reviewed iteratively, and participant feedback was organised around key domains: interpretation, cultural relevance, and linguistic clarity (Guillemin et al., 1993; Sousa and Rojjanasrirat, 2011). These domains proposed item modifications.

Together, the item-level comparative review and qualitative analysis of focus groups enhanced linguistic precision and strenghtened the cultural validity of the adapted tools.

### 2.5 Research procedure

The cultural adaptation process typically begins with forward translation into the target language (e.g., Cruchinho et al., 2024; Snider and Schafer, 2018; Zhao et al., 2024). However, as Slovak versions of the selected tools were already in use, the study worked with the existing translations.

The primary aim was to refine these versions to ensure linguistic clarity and cultural validity. Although substantial data had already been collected using existing versions, the research-practice partnership team remained open to revisions informed by empirical findings from the qualitative phase.

The process began in February 2025, when the research-practice partnership team identified items for qualitative exploration based on practitioner feedback from meetings of the Koalicia skol za dusevne zdravie. In parallel, the first author reviewed the Slovak translations against the original versions and consulted practitioners regarding previous translation decisions. Items requiring further exploration were prioritised, while remaining open to new issues and suggestions raised by the participating children and young people during revised questionnaire administration and focus group discussions.

In March and April 2025, two consecutive teaching sessions (90 min in total), were allocated per group. Six groups completed the questionnaires and then participated in focus group discussions. Focus groups were audio-recorded using an offline voice recorder and documented in researcher notes. Based on participant feedback, the first author revised selected items, and the updated version was shared with the research-practice partnership team before back-translation.

In May 2025, back-translation into English was conducted by an independent translator with no prior involvement. The first author and translator reviewed discrepancies and agreed on final revisions to the Slovak versions.

In June 2025, version 2.0 of the selected tools was finalised and issued. The specific changes are outlined in the Result section.

### 2.6 Ethical considerations

Ethical approval was obtained from the Ethics committee, Faculty of Social and Economic Studies, Comenius University in Bratislava, Slovakia. The study was conducted in accordance with the Declaration of Helsinki, GDPR regulations under the Act No. 18/2018, and the ethical guidelines of the University.

Given that participants were under 18 years of age, informed consent was obtained from parents or guardians and head teachers. Young people aged 16 and over also provided consent, while younger participants provided assent. Age appropriate information sheets and consent forms were distributed.

Participants were informed verbally and in writing about the study’s purpose, voluntary participation, and their right to withdraw. They were also provided with information about available mental health support services.

At the end each session, participants were invited to share reflections on the process and to provide any feedback.

## 3 Results

This section presents the findings from the cross-cultural adaptation process. Across adaptation phases, only a small number of item-level modifications were identified for the Me and My Feelings scale, the Student Resilience Survey, and the Short Warwick–Edinburgh Mental Wellbeing Scale. To support readability, Slovak item wording is presented only where it is essential to the linguistic, semantic, or cultural analysis of questionnaire items; elsewhere, English-only wording is used.

The initial phases focused on reviewing and revising selected tools. Proposed modifications to the existing Slovak versions were documented in a summary list and annotated within the questionnaire documents. These were reviewed and discussed within the research-practice partnership team, resulting in a finalised list of items to be explored with participating children and young people.

To ensure that adaptations did not alter the underlying constructs, selected modifications, including items with potential conceptual implications and the revised response format, were shared with the original authors and the Anna Freud Centre for transparency and optional feedback. Although no further discussions took place and formal approval was not required, this step supported conceptual integrity and good practice in cross-cultural adaptation. Overall, the number of proposed changes was low across all tools. We first summarise participants’ experiences and researchers’ observations from administering the questionnaires. The Results sections follow the order of the study objectives.

### 3.1 Acceptability and feasibility of administering and completing the questionnaires

We systematically documented challenges observed during administration and identified issues related to (a) administration and technical feasibility, (b) item clarity and comprehension, and (c) completion time and response processing. Prior to administration, participants were informed that responses were anonymous and that there were no right or wrong answers. They were invited to raise their hand if they had questions. Only a small number did so. Overall, participants were willing to engage with the questionnaires and actively discuss their content during the subsequent focus groups.

#### 3.1.1 Administration and technical feasibility

At the upper levels (Year 10 or 2nd year of grammar school), questions primarily concerned technical access, including uncharged tablets, unstable internet connection, and difficulties accessing the questionnaire via QR code. The QR code, generated using a free online tool, required navigation through advertisements, which led to confusion. For example, one participant stated, *“I am not sure where to click now, there is so much advertisement”* (Girl1, Year10, Gymnasium), while another asked, *“Where is the questionnaire? Where does it start?”* (Boy1, Year8).

To mitigate feasibility challenges, we provided hard copies for primary pupils as not all had access to tablets, computers, or mobile phones. Printed backups were also prepared for lower and upper secondary school students and were used when occasional internet connectivity issues occurred. A few participants expressed a preference for completing the paper version.

During primary-level administration, class teachers remained present without a formal role. This decision was made to support pupils’ psychological comfort, given their established relationship with the teacher.

#### 3.1.2 Item clarity and comprehension

Requests for clarification were most frequent at the primary level and primarily concerned item wording. At lower and upper secondary levels, clarifications were less common but still occurred.

Five pupils from a Ukrainian background requested clarification of items including *“I feel lonely” and “I help my family make decisions.”* We examine these items in greater detail in the next section.

#### 3.1.3 Completion time and response processing

Primary school pupils required the longest time to complete the questionnaires, with substantial individual differences. In Year 3 at Site 1, most pupils completed the questionnaires within approximately 20 min. However, three pupils required considerably longer, with the last pupil finishing after 43 min. A similar pattern was observed in Year 4 at Site 2. In both cases, extended completion times were associated with reading difficulties.

Although five pupils from a Ukrainian background requested clarification of selected items, their overall completion times were comparable to their Slovak peers. Pupils who finished earlier remained patient and engaged in quiet reading, while waiting for others to complete the task.

**Figure 1:**
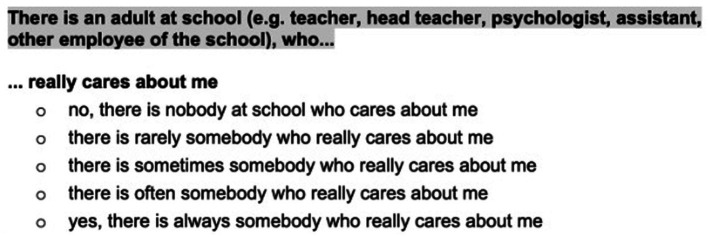
Sample back-translated item from the student resilience survey.

Perceptions of questionnaire length were mixed. Some participants considered the length appropriate, while others commented that the response options were long. During focus group discussions, when prompted, several participants acknowledged that they did not read all response options fully. This appeared acceptable, as the key meaning of the Likert-scale categories were conveyed in the initial wording of each option (see Figure 1).

#### 3.1.4 Overall acceptability

Most participating children and young people reported little or no previous experience with completing questionnaires, particularly at the primary and lower secondary levels. Some upper secondary students reported previous exposure to surveys, although generally on different topics. One participant reflected, “One *of our teachers in my previous school in [name of the location] used to hand us similar questionnaires about how we are, so yeah, I am used to filling these in”* (Girl1, Year 8). However, that was not one of the tools used in the present study.

In the final evaluation of the session, no participants reported significant difficulties or negative attitudes towards the activity. Responses such as *“It was okay”* (Boy1, Year6) and *“easy”* (Boy1, Year8) were typical. The creative group activity introduced at the end of the discussion (poster development on calmness, success, and connectedness) appeared to contribute positively to the overall experience, with several groups expressing interest in displaying their posters in their classrooms.

### 3.2 Understanding and interpreting the questionnaires’ items

To identify questionnaire items for group discussions, we drew on the research-partnership team’s prepared item list, researcher notes on questions raised during administration, and open-ended questions to participants about any words that seemed “strange or funny.” Findings are organised into three analytic areas: abstract concepts, linguistic and semantic equivalence, and cultural a.

#### 3.2.1 Abstract concepts

During administration, primary school pupils most often asked about abstract concepts. These included *“I feel lonely” [Cítim sa osamelo]* from Me and My Feelings questionnaire (Deighton et al., 2013) and *“I help my family make decisions”* from the Student Resilience Survey (Lereya et al., 2016). After exploring the understanding of this concept across all school levels, it seemed that only children from Ukraine and those with learning difficulties struggled to fully grasp the meaning of *“lonely”* and *“decisions.”* Older children and young adults found the expressions accurate and suggested using *“my parents ask me”* (Girl1, Year6) when explaining *“decisions”* to younger pupils, and *“feeling sad or missing friends when they are not around”* (Boy2, Year8) for feeling *“lonely.”* These suggestions were incorporated into the administrator manual to support explanation of abstract concepts, while retaining the original questionnaire wording.

We also explored *“I lose my temper” [Neovládam sa]* in Me and My Feelings (Deighton et al., 2013). Children provided concrete examples: *“That’s when I scream”* (Boy1, Year3), “I scatter my toys” (Boy2, Year3), and *“When someone loses their temper, they do not listen to the teacher”* (Girl1, Year4), confirming comprehension across participants.

Lower secondary students were initially uncertain about *“Are there Students at your School...who would miss you if you were not at school’* from the Student Resilience Survey (Lereya et al., 2016). With these students and young adults, we explored how this item related to their everyday experiences. In discussion, participants clarified using real-life examples (all names are pseudonymised):

Author1: *Is there anyone who would miss you if you were not at school?*

Boy1, Year10, Industrial school of engineering: *Yes, Mike would.*

[Group laughs; Mike nods in agreement]

Author1: *How do you know he would miss you?*

Boy2, Year10, Industrial school of engineering: *Because he tells us so*

Author1: *Really? That's so nice.*

Boy2, Year10, Industrial school of engineering: *..and he also texts us that he has no one to talk to in school.*

Similar responses appeared in other schools: *“He texts me ‘where are you? Why are you home? Are you faking it again?’ He must be missing me then.”* (Boy1, Year10, Gymnasium), and *“We WhatsApp each other if one of us does not arrive to school”* (Girl1, Year8). These examples may help administrators illustrate item meaning if students seek clarification.

#### 3.2.2 Linguistic and semantic equivalence

Asking participants for specific examples was effective across other items. For instance, ‘*At School, there is an adult who... ... believes that I will be a success*’ *[V škole je niekto, kto... verí, že budem mať úspech]* in the Student Resilience Survey (Lereya et al., 2016) prompted responses such as “a coach” (Girl1, Year10, Gymnasium). Similarly, ‘*I do things at home that make a difference (i.e., make things better) [Doma dokážem meniť veci k lepšiemu] and ‘I help my family make decisions’ [V rodine sa podieľam na rozhodnutiach*] elicited interpretations spanning personal and family contexts:

Author2: *How did you understand the item 'I can make things better at home'?*

Boy1, Year10, Gymnasium *:Whether I am becoming a better person myself at home.*

Author2: *Mhm, whether you are becoming a better person at home...*

Boy1, Year10, Gymnasium *:Well that, but also overall in the family, and also everything else that relates to home. I think it was clear.*

Author2: *What about the rest of you? Were you clear about the wording of the item?*

Girl2, Year10, Gymnasium: *Not really.*

Author2: *Not really*

Girl2, Year10, Gymnasium: *It is a strange item really.*

Author2: *Strange? In what way?*

Girl2, Year10, Gymnasium: *What are the things to make better? There may be more things*

Author1: *Those of you who did not find it strange, what things or situations did you think of in terms of you being able to improve at home?*

Girl3, Year10, Gymnasium: *To make amends after having an argument*

Children and young people repeatedly mentioned *“cleaning, helping with the houses chores”* (e.g., Boy3, Year10, Industrial school of engineering) as examples of ways to make a difference at home. A couple of older participants noted *“improving relationships”* (Boy2, Year10, Industrial school of engineering), *“financially”* (Boy4, Year10, Industrial school of engineering) and one stated *“waste separation”* (Girl2, Year4).

For the item *“I help my family make decisions,”* typical examples included *“choosing a holiday destination”* (Boy2, Year10, Gymnasium), *“where to go for a day trip”* (Girl2, Year10, Industrial school of engineering), and *“what meal to cook”* (e.g., Girl1, Year6). When we suggested choosing a television as an example, participants responded with laughter, explaining that families rarely watch television together in the living room. Instead, children and young people tend to watch online series on different streaming platforms in their own rooms. This exchange highlighted generational shifts in shared household activities.

There were also instances where anticipated linguistic difficulty was not confirmed by participants. For example, regarding the phrase *“I help make decisions” [podieľam sa*], one participant commented *“I think ‘help make decision’ is an ideal expression”* (Boy1, Year10, Gymnasium).

#### 3.2.3 Cultural adaptation

Some items required semantic or cultural clarification. For instance, the term *‘partner’* [partner] in in the item *“Are there Students at your School who would... pick you for a partner” [V škole sú žiaci a žiaci, ktorí... si ťa vyberú za partnera/partnerku (napr. pri úlohe alebo hre)]* could carry a romantic meaning. Participants noted: *“Yeah it did cross my mind, but when it says in a task, it is clear”* (Boy1, Year10, Gymnasium), or *“I think it’s okay. It [partner] is generally used”* (Girl2, Year10, Industrial school of engineering). Alternative suggestions, such as *“friend”* (Boy1, Year10, Gymnasium), or *“perhaps mate”* (Boy3, Year10, Industrial school of engineering), risked narrowing the meaning to someone with closer relationship to a child and were therefore not adopted.

The largest discussions arose around two items in the Student Resilience Survey, which required clearer delineation:

“*Away from school...*

*... I am a member of a club, sports team, church group, or other group*

*... I take lessons in music, arts, sports, or have a hobby” (Student Resilience Survey,* Lereya et al., 2016)

The Slovak translation in Pohodomer 1.0 stated:

*[Mimo školy...*

*... chodím na krúžky, som člen/členka mimoškolskej organizácie alebo klubu (základná umelecká škola, turitiska, skaut, kone, ochrana prírody a pod., športového tímu, cirkevného mládežníckeho spoločenstva (tzv. stretko), alebo inej podobnej skupiny*

*... chodím na krúžky (hudba, umenie, šport, alebo mám iného koníčka)].*

In Slovak, memberships and taking lessons often overlap because lessons are typically organised by established institutions that require memberships (e.g., art schools). Some participants commented: *“I answered the questions very similarly”* (Boy4, Year10, Industrial school of engineering), which aligned with the feedback we received from the Pohodomer administrators and indicated insufficient differentiation between the two items.

To address this, we strengthened the membership component in the first item, inspired by students’ examples such as being *“a member of an organisation, like the debating association”* (Boy2, Year 10, Gymnasium). For the second item, we retained the term *‘krúžky*,’ which literally refers to organised activities but does not necessarily imply formal membership. Participants proposed clarifying the meaning through examples such as *“free-time activities, like maybe drawing at home”* (Girl4, Year10, Industrial school of engineering), and suggested listing “Music, art, sport” (Year 10, Gymnasium) as illustrative activities. These suggestions were merged into the revised wording.

This consultation resulted in an updated Slovak version of the two items in Pohodomer 2.0:

*[Mimo školy...*

*... som člen/členka klubu, športového tímu, skautov, cirkevného mládežníckeho spoločenstva (tzv. stretko), alebo inej podobnej skupiny (napr. turistického krúžku)*

*.. chodím na krúžky (hudba, umenie, šport) alebo mám iné záľuby)].*

Back-translated to English, the item read:

*“Away from school...*

*... I am a member of a club, sports team, scouts, church youth fellowship (called a “meetup”), or other similar group (e.g., hiking club)*

*.. I take lessons (music, art, sports) or have other hobbies”*

Probing differences between items and eliciting concrete examples proved a valuable strategy for ensuring conceptual distinction and meaningful cultural adaptation.

We also asked for examples of individuals outside the family for the item section ‘*Away from School, there is an adult who.’ [Mimo školy či rodiny je dospelá osoba, ktorá.]* in the Student Resilience Survey*,* Lereya et al., 2016). Based on participants’ responses, we added examples such as *‘coach, interest group leader, an adult from my neighbourhood, family friends, and church or community member’* to the Slovak version to enhance clarity.

Response options also required consideration. In the Me and My Feelings questionnaire, the options include never-sometimes-always. In Slovak, the word always can be interpreted very rigidly, meaning “every time,” making children and young people less likely to select it even if the statement generally applies. Following internal discussion within Koalicia skol za dusevne zdravie, always was replaced *“often,”* which was considered more relatable and reflective of typical usage. Although a similar nuance exists in English, the effect appears particularly pronounced in Slovak.

Overall, discussions with children and young people confirmed understanding of the existing translations in Pohodomer 1.0 and generated valuable suggestions. Proposed revisions were discussed within the research-partnership team, reviewed during back-translation, and where necessary, further consulted with the translator. The original authors of the tools and the Anna Freud Centre were informed of the changes. No formal approval was required.

All suggestions from participating children and young people were taken into account. They were either incorporated into the new version, Pohodomer 2.0, or documented in the administrator manual for Pohodomer 2.0. Tables 4–6 summarise the item-level modifications made during the adaptation process from Pohodomer 1.0 to Pohodomer 2.0 for each measure.

**Table 4:** Comparison of item wording in Pohodomer 1.0 to Pohodomer 2.0: *Me and My Feelings* scale.

Original item	Pohodomer 2.0 version	Pohodomer 1.0 version	Back-translation of Pohodomer 1.0 version
5. I feel lonely	Cítim sa osamelo	Cítim sa sám/sama	I feel alone
12. I feel shy	Som hanblivý/á	Hanbím sa	I feel embarassed
14. I worry when I am at school	*V škole som nervózny / nervózna	V škole som nervózny / nervózna	I am nervous at school
18. I do things to hurt people	Robím veci, aby som ľuďom ublížil/a	Robím veci, ktoré ľuďom ubližujú	I do things which hurt othe people

^*^Pohodomer 1.0 version remains after discussing cultural validation.

**Table 5:** Comparison of item wording in Pohodomer 1.0 to Pohodomer 2.0: *Student Resilience Survey.*

Original item wording	Pohodomer 2.0 version	Pohodomer 1.0 version	Back-translation of Pohodomer 1.0 version
4. At home, there is an adult who... ... listens to me when I have something to say	Doma je niekto dospelý (rodičia, príbuzní, pestúni, opatrovníci...), kto, “...ma počúva, keď chcem niečo povedať”	Doma je niekto, kto “... vypočuje si ma, keď sa potrebujem porozprávať”	At home, there is someone who “...listens to me when I need to talk”
7. At School, there ia an adult who... ... listens to me when I have something to say	V škole je niekto dospelý (napr. učiteľ/ka, riaditeľ/ka, psychológ/ička, asistent/ka, iný zamestnanec/kyňa školy), kto…“...ma počúva, keď chcem niečo povedať”	V škole je niekto, kto “vypočuje si ma, keď sa potrebujem porozprávať”	At home, there is someone who “...listens to me when I need to talk”
13. Away from School... ... I am a member of a club, sports team, church group, or other group	Mimo školy... ... som člen/členka klubu, športového tímu, skautov, cirkevného mládežníckeho spoločenstva (tzv. stretko), alebo inej podobnej skupiny (napr. turistického krúžku)	Mimo školy... ... chodím na krúžky, som člen/členka mimoškolskej organizácie alebo klubu (základná umelecká škola, turistika, skaut, kone, ochrana prírody a pod.), športového tímu, cirkevného mládežníckeho spoločenstva (tzv. stretko) alebo inej podobnej skupiny	Away from school... I am a member of an extracurricular organisation or club (art school, hiking, scouts, horses, tourist club, environmental organisation, etc.), a sports team, a church youth group (so-called “meet-up”) or another similar group
14. Away from School...... I take lessons in music, arts, sports, or have a hobby	Mimo školy... ... chodím na krúžky (hudba, umenie, šport) alebo mám iné záľuby	Mimo školy... ... chodím na krúžky (hudba, umenie, šport, alebo mám iného koníčka)	Away from school.... I attend structured activities (music, art, sports), or I have another hobby.
22. Are there students at your School who would... make you feel better if something is bothering you	V škole sú žiaci a žiačky (či už v tvojej alebo inej triede), ktorí/é... ... pomôžu ti cítiť sa lepšie, ak ťa niečo trápi	V škole sú žiaci a žiačky (či už v tvojej alebo inej triede), ktorí/é... ... pomôžu ti cítiť sa lepšie, ak ťa niečo hnevá	At your School, there are students who would... ...make you feel better if you get annoyed
24. Are there students at your School who would...... help you if other students are being mean to you	V škole sú žiaci a žiačky (či už v tvojej alebo inej triede), ktorí/é... ... pomôžu ti, ak sú na teba iní spolužiaci/spolužiačky zlííif other students are being mean to you	V škole sú žiaci a žiačky (či už v tvojej alebo inej triede), ktorí/é... ... pomôžu ti, ak sa k teba iní spolužiaci/spolužiačky správajú zle	At your School, there are students who would .... ... make you feel better if other students behave badly
35. I feel bad when someone gets their feelings hurt	Je mi ľúto, keď má niekto zranené city	“Cítim sa zle, keď v mojom okolí niekomu ubližujú”	I feel bad when somebody around me is mistreated

**Table 6:** Comparison of item wording in Pohodomer 1.0 to Pohodomer 2.0: *Short Warwick–Edinburgh Mental Wellbeing Scale.*

Original item	Pohodomer 2.0 version	Pohodomer 1.0 version	Back-translation of Pohodomer 1.0 version
Start the answer sheet with a statement beginning with “In the past two weeks…” to remind students of the two-week period in the instructions and to reflect the meaning of the present perfect tense	“V posledných dvoch týždňoch....”	Not existing	Not existing
1. I’ve been feeling optimistic about the future	“...vidím budúcnosť optimisticky”	myslela som si, že v budúcnosti veci dobre dopadnú	I have thought things will turn out well in the future
10. I’ve been feeling close to other people	Cítim, že mám blízke vzťahy s inými ľuďmi	Cítil/a som sa dobre s inými ľuďmi	I have felt good with other people

## 4 Discussion

This study illustrates a research-practice partnership in the cultural validation of mental health screening tools for children and young people. It focused on the initial phase of adapting instruments for use in a different cultural context, with attention to translation and cross-cultural adaptation (e.g., Cruchinho et al., 2024; Sousa and Rojjanasrirat, 2011). To support this process, we established a collaboration between university researchers and a national mental health charity, aiming to bridge academic rigour with practical, school-based needs (McGeown and Sjölund, 2025).

Balancing practical constraints (e.g., limited expertise, time pressures, unstable internet access in schools) with structured research requirements (e.g., systematic item review, scripted meetings, adherence to ethical research protocols) created opportunities for mutual learning. Such tensions are commonly reported in research-practice partnerships in education, where differences in expertise, time, and institutional resources function both as barriers and as productive spaces for co-learning and methodological innovation (McGeown and Sjölund, 2025; Duea et al., 2022). Our findings extend this literature by illustrating how these dynamics manifest specifically in the cultural validation of school-based mental health screening tools.

We adopted a participatory approach by consulting children and young people across three educational levels to ensure developmental appropriateness and contextual relevance (Duea et al., 2022, International Collaboration for Participatory Health Research [ICPHR], 2013). Their direct experience completing the questionnaires and the feedback they provided substantially enhanced the quality of the translation and cultural adaptation. This aligns with participatory health research literature emphasising that involving end users, particularly children and young people, improves semantic clarity, relevance, and acceptability (ICPHR, 2013; Duea et al., 2022).

Importantly, participants did not merely confirm researchers’ assumptions about potentially unclear wording. In some instances, children and young people explicitly disagreed with our concerns and defended particular expressions as appropriate. This willingness to challenge the authors’ interpretations suggests that the discussion environment supported open dialogue and reduced authority bias, thereby strengthening the participatory nature of the process.

Consistent with evidence from focus group studies, children’s input was particularly valuable in identifying difficulties related to abstract or culturally unfamiliar concepts (Guillemin et al., 1998; Peña, 2007; Schoua-Glusberg and Villar, 2014; Snider and Schafer, 2018; Sousa and Rojjanasrirat, 2011). While relatively few comprehension difficulties emerged overall, formatting adaptations (e.g., listing full response options beneath each item) were perceived as manageable. Participants also proposed concrete examples that were incorporated into wording or the administrator manual, supporting clearer interpretation in future use.

Overall, experiences of questionnaire completion were largely neutral, with some participants perceiving the tools as lengthy and others not. Similar variability has been documented in large-scale school-based mental health studies, where engagement, language background, and educational needs shape perceived burden and acceptability (Otto et al., 2021; Smith et al., 2021). We observed notable individual differences, particularly in classrooms including children from non-Slovak language backgrounds or with special educational needs. These observations informed practitioner-facing recommendations emphasising the importance of appropriate support during questionnaire administration.

For most participants, this was their first experience of completing self-report mental health questionnaires, highlighting the limited integration of systematic mental health screening within the Slovak education system. Internationally, regular mental health monitoring is increasingly embedded within public health and education frameworks, as reflected in initiatives such as the Health Behaviour in School-aged Children study and the use of brief wellbeing measures [World Health Organisation (WHO) 2024, 2025]. The absence of standardised screening tools in Slovak schools therefore represents a significant gap that culturally validated instruments could help address. Although plans to introduce regular school-based mental health monitoring are emerging, the Ministry of Education and its affiliated organisations have not yet introduced standardised tools for this purpose. Insights from this study, alongside the potential adoption of the culturally validated instruments, may offer a practical starting point for advancing school-based mental health screening in Slovakia and in other contexts adapting tools developed in English-speaking settings.

Mental health questionnaires not only generate data on students’ psychological wellbeing and distress but also elevate conversations about mental health within the school environment. They introduce a shared “mental health vocabulary” into settings where stigma remains prevalent and equip educators, support staff, and school leaders with structured means to address mental health proactively. Their presence may also signal to students and parents that the school takes mental health seriously.

To achieve cultural equivalence Me and My Feelings (Deighton et al., 2013), Student Resilience Suvey (Lereya et al., 2016), and SWEMWBS (Stewart-Brown et al., 2009; Warwick-Edinburgh Mental Well-Being Scale, 2008), we implemented targeted enhancements to the linguistic, semantic, cultural, and operational equivalence (Herdman et al., 1998; Peña, 2007). Particular attention was given to abstract concepts, such as loneliness, as well as culturally specific references (e.g., club memberships versus structured extracurricular activities). These challenges are consistent with theoretical models of cultural equivalence, which emphasise that abstract psychological constructs are especially vulnerable to semantic and conceptual mismatch across cultural contexts (Herdman et al., 1998; Peña, 2007). Our findings further align with qualitative evidence demonstrating culturally variable understandings of core wellbeing concepts, underscoring the importance of context-sensitive adaptation rather than direct or literal translation (Mao et al., 2021).

To address these issues, we worked collaboratively with participating children and young people to explore culturally equivalent forms of engagement in clubs, hobbies, and sporting or artistic activities within the Slovak context. Participants also helped identify linguistic and semantic equivalences and establish shared meanings, in line with universalist approaches to cultural adaptation (Herdman et al., 1998; Peña, 2007). One effective strategy involved incorporating concrete examples to illustrate abstract concepts, such as identifying supportive adult figures outside the home. As reported in the Cultural adaptation section of the Results (3.2.3), this approach was applied to the item “Away from school there is an adult who.,” with participants suggested examples including interest group leaders, family friends, neighbours, and other community members. This process illustrates the value of focus group discussions in uncovering culturally unfamiliar or abstract concepts and assessing item clarity and relevance (Guillemin et al., 1998; Peña, 2007; Schoua-Glusberg and Villar, 2014; Snider and Schafer, 2018; Sousa and Rojjanasrirat, 2011).

We also revised the operational equivalence (Herdman et al., 1998), particularly the presentation and wording of response options. Rather than retaining the original table-based formatting, items and corresponding responses were presented individually to encourage full reading and reflection by children and young people. In the Student Resilience Survey (Lereya et al., 2016), response options were displayed beneath each item as full-sentence statements, reducing the need for respondents to infer the meaning of abstract Likert-scale categories. This adaptation was intended to support comprehension among participants with limited prior exposure to self-report mental health questionnaires and to minimise the risk of superficial responding associated with dense tabular formats. Such modifications align with cross-cultural validation guidelines prioritising operational equivalence and respondent understanding over strict adherence to original formatting (Guillemin et al., 1993; Sousa and Rojjanasrirat, 2011), as well as applied recommendations for questionnaire design with younger respondents (Snider and Schafer, 2018).

A recognised limitation of presenting full-sentence response options is the resulting increase in questionnaire length. Although many lower and upper secondary participants reported quickly becoming familiar with the Likert-scale structure and not always reading each option in full, observational data from primary school pupils indicated variability in reading fluency and completion times. In addition, consultation with Coalition administrators who had prior experience administering earlier versions of the questionnaire empdhasise the importance of maintaining clearly worded response options to support diverse learners. Taken together, these considerations informed our decision to retain the expanded response format while visually emphasising key Likert-scale terms to facilitate more efficient navigation.

As part of the translation and cultural validation process, we have reached out to the original authors of the tools and the Anna Freud Centre. We outlined the formatting and wording changes that extended beyond literal translation. For example, items in Me and My Feelings *‘I feel scared’ [Mám strach]* and ‘I worry when I am at school’ *[Mám strach/obavy, keď som v škole]* (Deighton et al., 2013) were initially translated using overlapping Slovak terms conveying fear or apprehension. To better differentiate the underlying constructs, we proposed using the word *‘nervous’* in place of *‘worry’,* reflecting a more natural distinction in Slovak usage. This approach illustrates our strategy of prioritising symmetrical translation to preserve conceptual meaning and cultural relevance of the tools rather than relying on literal equivalence (Cruchinho et al., 2024; Sousa and Rojjanasrirat, 2011). Similar strategies have been applied in other cross-cultural validation studies, including adaptations conducted in Sweden (Granat et al., 2022).

Consistent with Hawkins et al. (2020), we conceptualised translation decisions as a source of validity evidence, demonstrating how symmetrical translation can support construct equivalence in self-reported mental health measures. By transparently documenting these decisions and their rationale, this study contributes to the growing body of literature on rigorous, context-sensitive validation of psychological instruments beyond English-speaking settings, in line with foundational principles of cross-cultural research (Brislin, 1970).

### 4.1 Reflecting on research-practice partnership

This study was shaped by a research-practice partnership that combined everyday school experiences with research rigor. This collaboration guided decisions and ensured that cultural adaptations were systematically documented and reflected upon, creating a stronger foundation for transparency and future development. As highlighted by recent literature (McGeown and Sjölund, 2025), partnerships of this kind are essential for developing tools that are both methodologically sound and relevant for real-world contexts. Shared expertise between researchers and practitioners enhanced alignment with practical needs in schools and laid the groundwork future cross-cultural validation, field testing, and full psychometric validation of the tools (Cruchinho et al., 2024). These reflections highlight how sustained collaboration between research and practice can strengthen the development and implementation of school-based mental health screening tools, contributing to meaningful educational change.

### 4.2 Recommendations for screening administrators

Our experience administering the selected tools, along with follow-up discussions, revealed several practical considerations for administrators and practitioners. A key issue is that not all schools, children, and young people in Slovakia have consistent access to the internet or digital devices such as mobile phones and tablets. This reflects broader infrastructural challenges, including regulations restricting mobile phones use in primary and lower secondary schools (Ministry of Education, Research, Development and Youth of the Slovak Republic and Research Institute of Child Psychology and Pathopsychology, 2024), as well as inconsistent internet connectivity, even in urban areas. These findings highlight structural limitations in digital access within the Slovak education system, which may similarly affect other countries implementing school-based mental health screening tools.

One output of this study was the development of revised recommendations for the Pohodomer Administrator Manual to support the standardisation of administration procedures and enhance the psychometric robustness of the tool. The manual is designed to ensure consistent administration and to provide all children and young people with the same information, particularly regarding anonymity and the absence of right or wrong answers. The Slovak education system places strong emphasis on knowledge-based assessment, which may encourage children to seek the ‘correct’ response or attempt to please teachers. This tendency may be particularly pronounced given that many pupils are unfamiliar with self-report questionnaires.

We found it essential to emphasise the importance of supporting comprehension of abstract concepts, especially among young participants, those from non-Slovak-speaking backgrounds, and children and young people with reading difficulties or special educational needs. This need is increasingly relevant in the Slovak context, given the recent arrival of children and young people from Ukraine and other regions affected by conflict. While teachers, who typically act as administrators, are generally familiar with their pupils’ individual needs, external administrators such as school psychologists or newly appointed teachers, may require additional guidance. Key recommendations are outlined in Appendix A. These practical considerations underline the importance of contextual feasibility when implementing school-based mental health screening tools, particularly in education systems with strong assessment cultures and variable digital infrastructure.

### 4.3 Strengths and limitations

A distinctive aspect of this study is the sequence in which translation and cultural validation were conducted. Typically, these precede implementation; however, in this case, the tools were already in use when the need for a more systematic, research-based approach became evident. Although this post-hoc validation is not methodologically ideal, Pohodomer version 1.0 had demonstrated practical value. Ongoing discussions between Koalicia skol za dusevne zdravie and member schools supported teachers and school psychologists in interpreting results withing their specific contexts and identifying areas requiring additional mental health and wellbeing support.

A key strengths of this study is the close collaboration between researchers and practitioners working directly with schools. This partnership grounded the adaptation process in real-world educational settings, enhancing practical relevance and usability. Another strength is the involvement of children and young people from diverse school types and regions across Slovakia, increasing contextual sensitivity and applicability.

Despite these strengths, several limitations should be acknowledged. The post-hoc nature of validation means that further psychometric testing is required, particularly for broader or longitudinal use. Although children and young people were actively involved, their participation was constrained by time, resources, and school-based practicalities. Future studies should include larger and more diverse samples and, where possible, conduct validation earlier in the adaptation process.

Another limitation relates to focus group discussions. While some items were prioritised based on earlier review phases, participants were invited to raise additional unclear terms, and clarification requests were documented during administration (e.g., difficulties with the term “lonely”). However, due to time constraints and the length of the questionnaires, a full item-by-item review was not feasible. Consequently, some potentially confusing elements may not have been identified or verbalised. Future studies could allocate more time or incorporate a systematic one-by-one exploration of all items to further strengthen participant-led input.

## 5 Conclusion

This study demonstrates three essential components in adapting mental health screening tools for school settings: (1) a research-practice partnership, (2) rigorous translation and cultural adaptation, and (3) a participatory approach involving children and young people.

Drawing on established models of cultural equivalence (Peña, 2007; Herdman et al., 1998) and the framework proposed by Cruchinho et al. (2024), we undertook a systematic review and refinement of the Slovak versions of *Me and My Feelings*, the *Student Resilience Survey*, and *Short Warwick-Edinburgh Mental Well-being Scale.* Feedback from pupils across three school levels revealed areas of ambiguity, particularly in relation to abstract or culturally specific concepts. This resulted in revisions to wording, examples, and formatting to enhance clarity and cultural relevance. Although these modifications increased the length of some items, navigational clarity was supported by visually emphasising Likert-scale keywords.

The participatory process highlighted that, for many children, this was their first experience completing self-report mental health questionnaires. Although the study excluded participants who had previously completed the specific screening tools under review, the findings point to a broader absence of systematic mental health screening practices within Slovak schools. The research-practice partnership between academic researchers and a national mental health organisation was central to balancing methodological rigour with contextual realities, including limited digital access and time constraints within school timetables. The development of a Pohodomer Administrator Manual further addressed classroom-specific challenges, such as pupils’ tendency to seek “correct” answers and the need for explicit guidance, particularly for children with language barriers or special educational needs.

Overall, the study demonstrates that culturally responsive adaptation of mental health screening tools benefits from collaboration with practitioners and end users. Such an approach strengthens clarity and cultural relevance while supporting the feasible and context-sensitive implementation in Slovakia and potentially in other countries adopting tools originally developed in English-speaking contexts.

## Data Availability

The datasets presented in this article are not readily available because of the ethical and data protection guidelines. The anonymised raw data supporting the conclusions of this study will be made available by the authors upon reasonable request. Requests to access the datasets should be directed to lenka.janik.blaskova@uniba.sk.

## Appendix A: Key recommendations for Pohodomer Administrators

1. Follow the standardised instructions outlined in the Pohodomer Administrator Manual developed by Koalicia skol za dusevne zdravie.
2. Clearly explain the anonymous nature of the questionnaires using age-appropriate and accessible language, allowing sufficient time for questions.
3. Emphasise that there are no right or wrong answers and that children and young people should respond based on how they feel at the time of completion.
4. Facilitate access to the online questionnaire by printing and distributing the provided QR codes and customised URL link.
5. Ensure printed copies are available as a backup in case digital access is limited or unavailable.
6. Be mindful of linguistic diversity. Check in with children and young people from non-Slovak-speaking backgrounds to ensure they understand items containing abstract terms (e.g., “lonely”), as noted in the Pohodomer Administrator Manual.
7. After administration, verify participation numbers to ensure all students have completed the questionnaire (both printed and online formats). For printed versions, check that all responses have been marked for all items.
8. Where possible, provide time for reflection or a brief creative follow-up activity after completion. Sample activities are included in the Manual.

## References

[ref1] Brislin R. W. (1970). Back-translation for cross-cultural research. J. Cross-Cult. Psychol. 1, 185–216. doi: 10.1177/135910457000100301

[ref2001] Cruchinho P. López-Franco M. D. Capelas M. L. Almeida S. Bennett P. M. Miranda da Silva M. . (2024). Translation, cross-cultural adaptation, and validation of measurement instruments: A practical guideline for novice researchers. Journal of Multidisciplinary Healthcar 17, 2701–2728. doi: 10.2147/JMDH.S419714 PMC11151507 38840704

[ref2] Deighton J. Tymms P. Vostanis P. Belsky J. Fonagy P. Brown A. . (2013). The development of a school-based measure of child mental health. J. Psychoeduc. Assess. 31, 247–257. doi: 10.1177/0734282912465570, 25076806 PMC4107815

[ref3] Dempsey C. Devine R. Fink E. Hughes C. (2024). Developmental links between well-being, self-concept and prosocial behaviour in early primary school. Br. J. Educ. Psychol. 94, 425–440. doi: 10.1111/bjep.12654, 38114272

[ref4] Duea S. R. Zimmerman E. B. Vaughn L. M. Dias S. Harris J. (2022). A guide to selecting participatory research methods based on project and partnership goals. J. Particip. Res. Methods 3. doi: 10.35844/001c.32605 PMC9258244 35799626

[ref5] Fekar Gharamaleki F. Fathipour-Azar Z. (2025). Cross-cultural adaptation and validation of cognitive emotion regulation questionnaire: a systematic review. Front. Psychol. 15:1494665. doi: 10.3389/fpsyg.2024.1494665, 40176871 PMC11961981

[ref7] Goodman R. (1997). The strengths and difficulties questionnaire: a research note. J. Child Psychol. Psychiatry 38, 581–586. doi: 10.1111/j.1469-7610.1997.tb01545.x, 9255702

[ref7001] Granat L. Andersson S. Hadziabdic E. Brännström M. Sandgren A. (2022). Translation, adaptation, and validation of the Self‑efficacy in Palliative Care scale (SEPC) for use in Swedish healthcare settings. BMC Palliative Care 21. doi: 10.1186/s12904-022-00940-5 PMC8995693 35410328

[ref8] Guillemin F. Bombardier C. Beaton D. (1993). Cross-cultural adaptation of health-related quality of life measures: literature review and proposed guidelines. J. Clin. Epidemiol. 46, 1417–1432. doi: 10.1016/0895-4356(93)90142-n, 8263569

[ref9] Hawkins M. Cheng C. Elsworth G. R. Osborne R. H. (2020). Translation method is validity evidence for construct equivalence: analysis of secondary data routinely collected during translations of the health literacy questionnaire (HLQ). BMC Med. Res. Methodol. 20:130. doi: 10.1186/s12874-020-00962-8, 32456680 PMC7249296

[ref10] Herdman M. Fox-Rushby J. Badia X. (1998). A model of equivalence in the cultural adaptation of HRQoL instruments: the universalist approach. Qual. Life Res. 7, 323–335. doi: 10.1023/A:1024985930536 9610216

[ref11] Institute for Health Metrics and Evaluation (2025). 2021 Global Burden of Disease Study. Seattle, WA: University of Washington. Available online at: https://vizhub.healthdata.org/gbd-results/?params=gbd-api-2019-permalink/380dfa3f26639cb711d908d9a119ded2 (Accessed 03 June, 2025).

[ref12] International Collaboration for Participatory Health Research (ICPHR). (2013). Position Paper No. 1. Available online at: http://www.icphr.org/position-papers--discussion-papers/position-paper-no-1 (Accessed 14 June, 2025).

[ref6] Janik Blaskova L. Winter L. (2025). Mapping teacher wellbeing in Slovakia: insights into resilience, relationships, and support needs. Front. Educ. 10:1584696. doi: 10.3389/feduc.2025.1584696

[ref13] Jones P. S. Lee J. W. Phillips L. R. Zhang X. E. Jaceldo K. B. (2001). An adaptation of Brislin’s translation model for cross-cultural research. Nurs. Res. 50, 300–304. doi: 10.1097/00006199-200109000-00008, 11570715

[ref14] Lereya S. T. Humphrey N. Patalay P. Wolpert M. Böhnke J. R. Macdougall A. . (2016). The student resilience survey: psychometric validation and associations with mental health. Child Adolesc. Psychiatry Ment. Health 10, 1–15. doi: 10.1186/s13034-016-0132-5 27822304 PMC5093941

[ref15] Mao Z. Ahmed S. Graham C. Kind P. Sun Y.-N. Yu C.-H. (2021). Similarities and differences in health-related quality-of-life concepts between the east and the west: a qualitative analysis of the content of health-related quality-of-life measures. Value Health Regional Issues 24, 96–106. doi: 10.1016/J.VHRI.2020.11.007, 33524902

[ref16] McGeown S. Sjölund S. (2025). Research-practice partnerships in education: benefits, challenges, methodological considerations and key enablers for change. Br. J. Educ. Psychol. 96, 1–13. doi: 10.1111/BJEP.12785, 40439951 PMC12879518

[ref17] Ministry of Education, Research, Development and Youth of the Slovak Republic and Research Institute of Child Psychology and Pathopsychology (2024). Guidance on changes to legislation regarding the use of mobile phones and similar personal electronic communication devices in schools [Usmernenie k zmene legislatívy ohľadom používania mobilných telefónov a obdobných osobných zariadení elektronickej komunikácie na školách]. Available online at: https://www.minedu.sk/data/att/4dd/31672.0c6aa4.pdf (Accessed 03 June, 2025).

[ref18] Otto C. Reiss F. Voss C. Wüstner A. Meyrose A. K. Hölling H. . (2021). Mental health and well-being from childhood to adulthood: design, methods and results of the 11-year follow-up of the BELLA study. Eur. Child Adolesc. Psychiatry 30, 1559–1577. doi: 10.1007/S00787-020-01630-4, 32918625 PMC8505294

[ref19] Peña E. D. (2007). Lost in translation: methodological considerations in cross-cultural research. Child Dev. 78, 1255–1264. doi: 10.1111/J.1467-8624.2007.01064.X, 17650137

[ref20] Rahman A. Iqbal I. Z. Waheed W. Hussain N. (2003). Translation and cultural adaptation of health questionnaires. J. Pak. Med. Assoc. 53, 142–147, 12776898

[ref21] Ronzón-Tirado R. C. Muñoz-Rivas M. J. Zamarrón Cassinello M. D. Redondo Rodríguez N. (2019). Cultural adaptation of the modified version of the conflicts tactics scale (M-CTS) in Mexican adolescents. Front. Psychol. 10:619. doi: 10.3389/fpsyg.2019.00619, 30949109 PMC6437088

[ref22] Schoua-Glusberg A. Villar A. (2014). “Assessing translated questions via cognitive interviewing,” in Cognitive Interviewing Methodology, eds. Miller K. Willson S. Chepp V. Padilla J.-L. (pp. 51–67). Hoboken, USA.

[ref23] Smith N. R. Marshall L. Albakri M. Smuk M. Hagell A. Stansfeld S. (2021). Adolescent mental health difficulties and educational attainment: findings from the UK household longitudinal study. BMJ Open 11:e046792. doi: 10.1136/bmjopen-2020-046792, 34305046 PMC8372813

[ref24] Snider L. Schafer A. (2018). Brief on Translating and Adapting the Psychological First Aid: Guide for field Workers. Peace in Practice and World Health Organization. Available online at: https://pscentre.org/wp-content/uploads/2018/12/2018-PFA-Translation-and-Adaptation-Guidance.pdf (Accessed 04 June, 2025).

[ref25] Sousa V. Rojjanasrirat W. (2011). Translation, adaptation and validation of instruments or scales for use in cross-cultural health care research: a clear and user-friendly guideline. J. Eval. Clin. Pract. 17, 268–274. doi: 10.1111/j.1365-2753.2010.01434.x 20874835

[ref26] Stewart-Brown S. Tennant A. Tennant R. Platt S. Parkinson J. Weich S. (2009). Internal construct validity of the Warwick-Edinburgh mental well-being scale (WEMWBS): a Rasch analysis using data from the Scottish health education population survey. Health Qual. Life Outcomes 7. doi: 10.1186/1477-7525-7-15, 19228398 PMC2669062

[ref27] Verde-Avalos E. Turpo-Chaparro J. E. Palomino-Ccasa J. Requena-Cabral G. Malca-Peralta S. S. (2025). Validation of measurement scale for technostress in Peruvian university students. Front. Psychol. 16:1503442. doi: 10.3389/fpsyg.2025.1503442, 40171077 PMC11959050

[ref28] Warwick-Edinburgh Mental Well-Being Scale. (2008). WEMWBS. © NHS Health Scotland, University of Warwick and University of Edinburgh. Available online at: https://warwick.ac.uk/fac/sci/med/research/platform/wemwbs/about/wemwbsvsswemwbs/ (Accessed 03 June, 2025).

[ref29] World Health Organisation (WHO). (2024). The World Health Organization-Five Well-Being Index (WHO-5). Available online at: https://cdn.who.int/media/docs/default-source/mental-health/who-5_english-original4da539d6ed4b49389e3afe47cda2326a.pdf?sfvrsn=ed43f352_11&download=true (Accessed 10 June, 2025).

[ref30] World Health Organisation (WHO). (2025). Health Behaviour in School-aged Children (HBSC): About. Available online at: https://hbsc.org/about/ (Accessed 10 June, 2025).

[ref7002] Zhao Y. Summers R. Gathara D. English M. (2024). Conducting cross‑cultural, multi‑lingual or multi‑country scale development and validation in health care research: a 10‑step framework based on a scoping review. Journal of Global Health 14:4151. doi: 10.7189/jogh.14.04151 PMC11257704 39024643

